# Label-Free Quantification of MicroRNAs Using Ligase-Assisted Sandwich Hybridization on a DNA Microarray

**DOI:** 10.1371/journal.pone.0090920

**Published:** 2014-03-10

**Authors:** Taro Ueno, Takashi Funatsu

**Affiliations:** Graduate School of Pharmaceutical Sciences, The University of Tokyo, Tokyo, Japan; The Scripps Research Institute, United States of America

## Abstract

MicroRNAs (miRNAs) can be used as biomarkers for cancer and other human diseases; therefore, high-throughput and reliable miRNA-quantification methods are required to exploit these markers for diagnostic testing. In this report, we describe the construction of a platform for miRNA-quantification using ligase-assisted sandwich hybridization (LASH) without miRNA-labeling. T4 DNA ligase was used to compensate for the low affinity between miRNAs and two short complementary DNA probes, and it improved the hybridization yield ∼50,000 times. The LASH assay enabled synthesized miR-143 to be quantified at concentrations ranging from 30 fM to 30 pM. The LASH assay could also quantify endogenous miR-143 released from cultured cells as well as some miRNAs in total RNAs derived from blood. Furthermore, multi-color detection enabled us to distinguish between the highly homologous miR-141 and miR-200a. This simple label-free quantification technique is an easy-to-use approach that can be applied to disease diagnosis.

## Introduction

MicroRNAs (miRNAs) constitute a class of small noncoding RNAs (typical length ∼22 nucleotides) that have been identified in the last decade. They play important roles in physiological processes, including proliferation, cardiogenesis and apoptosis, while miRNA dysfunction can bring about pathological processes such as tumorigenesis [1]–[3]. miRNAs are initially transcribed as primary precursor molecules (pri-miRNAs) from different genomic locations by RNA polymerase II [4]. Pri-miRNAs are processed by Drosha, a member of the RNAase-III family, to ∼70 nucleotides precursors called pre-miRNAs. Finally, the pre-miRNAs are cleaved to generate mature ∼22 nucleotides miRNAs [5]. The binding of mature miRNAs to 3′ untranslated regions of target mRNAs prevents their translation and reduces levels of the protein products [6]. Therefore, variations in miRNA expression levels are associated with various human diseases [7], [8]. In addition, miRNAs are present in blood serum, being protected from endogenous RNase activity, and the expression levels of some miRNAs are dysregulated in the serum of cancer patients [9]. Therefore, circulating miRNAs in blood serum are emerging as novel diagnostic and therapeutic targets for cancer and other human diseases [10].

High-throughput and reliable quantification of miRNAs is an essential step to enable their use in the diagnosis of diseases. To date, three primary technical platforms for miRNA expression profiling have been developed: qRT-PCR [3], deep sequencing [11] and DNA microarray technology. qRT-PCR covers a wide dynamic range and is usually used as the ‘gold standard’ for gene expression analysis, while deep sequencing has an advantage of identifying unknown miRNAs. However, the amplification processes required for these two technologies can lead to inherent biases. In addition, the throughput of qRT-PCR is generally low given the ever-increasing number of identified miRNAs, and deep sequencing requires a long runtime to generate an expression profile for each sample. On the other hand, DNA microarray technology can directly quantify miRNAs without amplification, and it is an efficient and economical method owing to its high-throughput performance. Currently, researchers can purchase commercial DNA microarrays from six companies [11]. However, most conventional microarrays, including all commercially available ones, require a miRNA pre-labeling process before quantification, despite it being time-consuming and expensive [11]. Furthermore the pre-labeling process tends to be liable to significant bias and artificial errors, thus non-labeling methods have been developed for direct quantification of miRNAs [12]. Currently, label-free technologies utilize enzyme-based colorimetric detection [13], dsRNA binding protein [14], stacking-hybridization [15], surface plasmon resonance [16], gold nano particle detection [17], electrochemical devices [18], surface-enhanced Raman spectroscopy [19], atomic force microscopy [20], exonuclease-based detection [21], and electrical switching devices [22]. Most of these methods are novel and have excellent potential, but suffer from difficulties of multiplicity, sensitivity, robustness, or economic viability that preclude extensive application to diagnostics.

A sandwich platform, based on a microarray where two kinds of probes bind to different sites on a target molecule, has the potential for high-throughput quantification of bio-molecules, including proteins and nucleic acids. This platform has significant advantages for high specificity, label-free detection and examples include, enzyme-linked immunosorbent assays for protein detection [23] and two-color microarray technology based on sandwich hybridization for nucleic acid detection [24], [25]. However, it is difficult to apply a sandwich platform to miRNAs because miRNAs are only ∼22 nucleotides long, which is too short for stable hybridization with two distinct probes, a fluorescent probe and a microarray-bound probe. Arata et al. reported a sandwich platform to quantify synthetic miRNAs, although the sensitivity was not sufficient to quantify endogenous miRNAs [26], [27].

**Figure 1:**
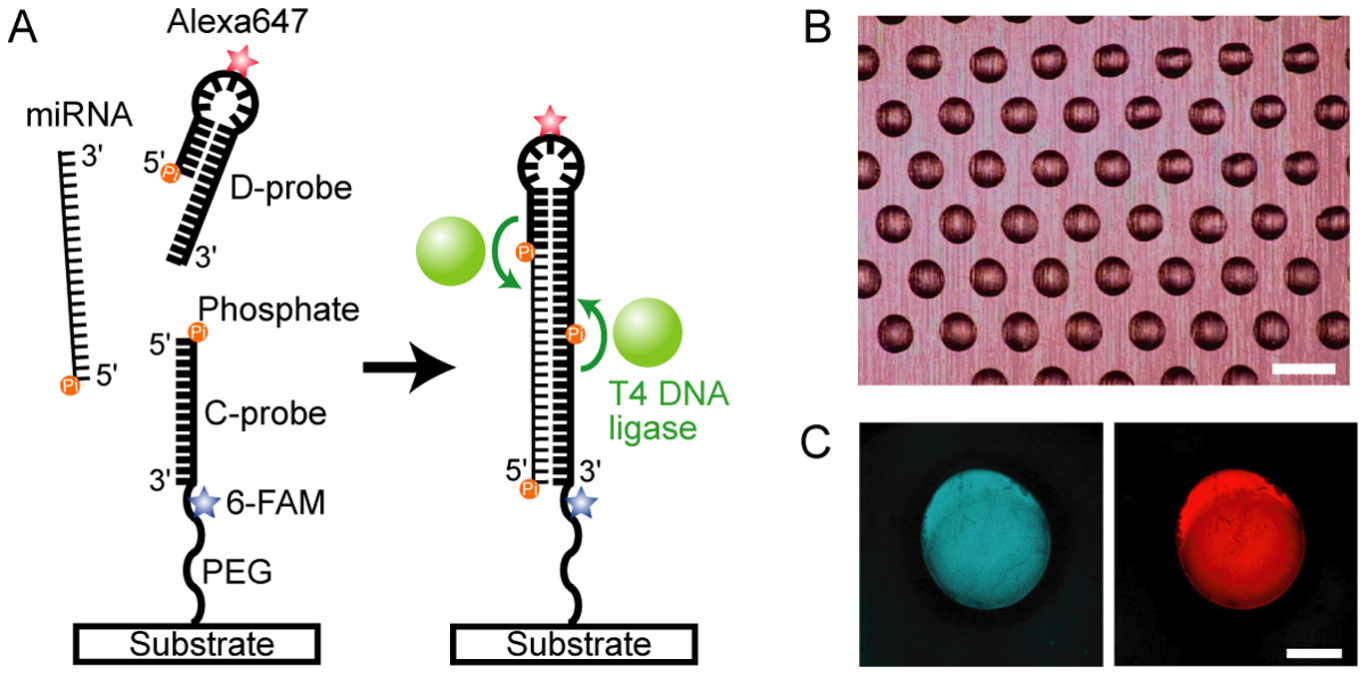
Experimental design of the LASH assay. (A) Schematic representation of the ligase-assisted sandwich hybridization assay (LASH assay) for detection of non-labeled miRNA. A target miRNA hybridizes to complimentary D-probe and C-probe, resulting in a tertiary complex. Both C-probe and D-probe are capped with a phosphate group at the 5′ end and a hydroxyl group at the 3′ end; this permits T4 DNA ligase to ligate the 5′ end of C-probe to the 3′ end of D-probe, and to ligate the 5′ end of D-probe to the 3′ end of the target miRNA. (B) A bright-field image of droplets spotted on a coverslip by an ink-jet machine. Scale bar, 400 µm. (C) Fluorescence images of 6-FAM-labeled C-probe (left) immobilized on a substrate and Alexa647-labeled D-probe (right) bound to C-probe in the presence of 1.25 nM miR-143. Scale bar, 100 µm.

Here we report on the development of a label-free DNA microarray system to quantify miRNAs based on a sandwich platform conjugated with a ligase-assisted reaction (Figure 1A). T4 DNA ligase is used to compensate for the low affinity between miRNAs and two separate complementary probes. This improves the hybridization yield ∼50,000 times, and we could quantify miR-143 at concentrations ranging from 30 fM –30 pM (10 amol –10 fmol in 0.3 ml of sample solution). This simple label-free quantification technique can be used for many applications, as well as for the development of miRNA quantification on a microfluidic chip, where it is difficult to label miRNAs with a fluorescent dye and then purify them.

## Materials and Methods

### Reagents

**Figure 2:**
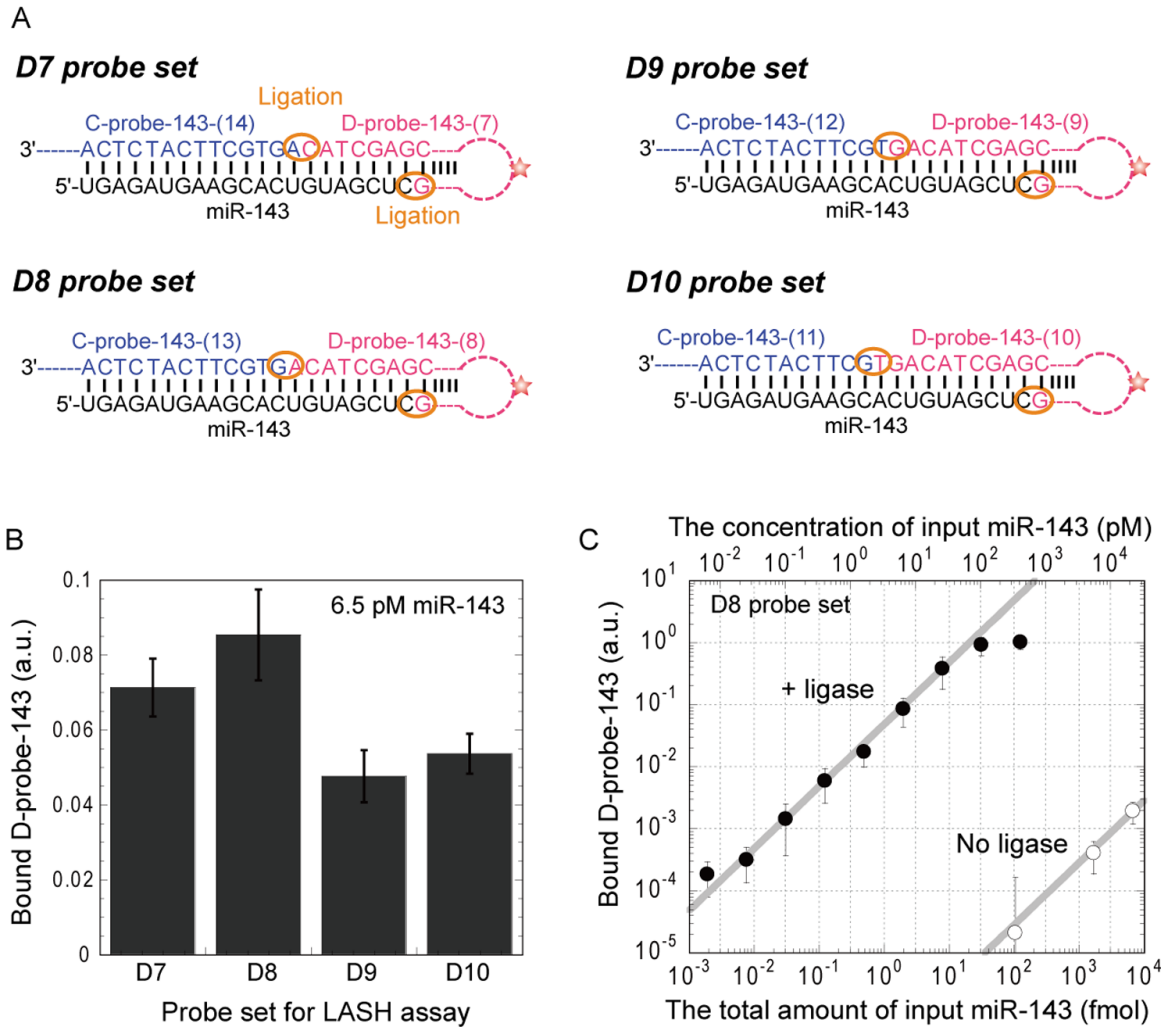
Optimization of D-probe complementary sequence length. (A) Complementary sequences of four probe sets for miR-143. (B) The fluorescence intensities of D-probes bound to C-probes in the presence of T4 DNA ligase. Data represent the mean ± S.E. (n = 3). (C) Correlation between input miR-143 and the signal of D-probe-143-(8) in the presence (filled circle) or absence (open circle) of T4 DNA ligase. The upper axis indicates the final concentration of input miR-143 in the hybridization chamber while the lower axis indicates the total amount of input miR-143. Data in the linear range were fitted by a linear expression (gray lines). Data represent the mean ± S.E. (n = 3).

Synthetic miRNAs were synthesized by Sigma Aldrich Japan (Tokyo, Japan). MiR-141, 5′-(UAA CAC UGU CUG GUA AAG AUG G)-3′, miR-143, 5′-(UGA GAU GAA GCA CUG UAG CUC)-3′, miR-21, 5′-(UAG CUU AUC AGA CUG AUG UUG A)-3′, miR-16, 5′-(UAG CAG CAC GUA AAU AUU GGC G)-3′, miR-92a, 5′-(UAU UGC ACU UGU CCC GGC CUG U)-3′ and cel-miR-39, 5′-(UCA CCG GGU GUA AAU CAG CUU G)-3′ were used as the target sequences. A passenger strand of miR-143, 5′-(GGU GCA GUG CUG CAU CUC UGG U)-3′ was used in negative control experiments. Oligonucleotides were obtained from Gene Design (Kyoto, Japan) or Eurofins (Kanagawa, Japan). For miR-143, four probe sets were prepared as follows. The thiolated DNA oligonucleotide, C-probe-143-(14), p-5′-(AGT GCT TCA TCT CA A CAA CAA CAA CAA CAA CAA CA)-3′-(6-FAM)-SH was used as a capture probe (termed C-probe). D-probe-143-(7), p-5′-(CTC AAC TGG TGT CGT GGA(-Alexa647) GTC GGC AAT TCA GTT GAG GAG CTA C )**-**3′ was used as a detection probe (termed D-probe) to detect miR-143. These two probes were used as the D7 probe set. Three other probe sets were prepared as in Fig. 2A. For miR-141, C-probe-141-(14), p-5′-(ACC AGA CAG TGT TA A CAA CAA CAA CAA CAA CAA CA)-3′-(6-FAM)-SH and D-probe-141-(8), p-5′-(CTC AAC TGG TGT CGT GGA(-Alexa647) GTC GGC AAT TCA GTT GAG CCA TCT TT )**-**3′ were prepared. For the two-color detection experiment, D-probe-200a-(8), p-5′-(CTC AAC TGG TGT CGT GGA(-Alexa532) GTC GGC AAT TCA GTT GAG TCA TCG TT )**-**3′ was prepared, while C-probe-141/200a-(14) was identical to C-probe-141-(14). Underlined sequences represent complementary sequences of target miRNAs. For miR-21, miR-16, miR-92a and cel-miR-39, D-probe-(8) was prepared as above. Silane-PEG-Maleimide (MW 5000), betaine solution, PEG 6000 solution, bovine serum albumin (BSA) and T4 DNA ligase were purchased from NANOCS Inc. (Boston, MA, USA), WAKO (Osaka, Japan), Hampton Research (Aliso Viejo, CA, USA), Sigma Aldrich Japan (Tokyo, Japan) and TAKARA (Shiga, Japan), respectively.

### Microarray Fabrication

Thiolated C-probes were reduced in buffer A [84 mM Tris-HCl, pH 8.0, 40 mM dithiothreitol (DTT)] for 1 h at room temperature and then DTT was removed using a NAP-5 column (GE Healthcare Biosciences, Piscataway, NJ, USA) equilibrated with buffer B (450 mM NaCl, 45 mM sodium citrate, pH 7.0). C-probes were then mixed with Silane-PEG-Maleimide reagent at the molar ratio of 1∶3 at room temperature in buffer B. After a 1-hour incubation, DTT (final concentration 10 mM) was added to quench the reaction. A printing solution was then prepared containing 3 µM C-probe-PEG-silane and 750 mM betaine in buffer B. A non-treated 18×18 mm coverslip (Matsunami, Osaka, Japan) was inserted on an ink-jet machine (LaboJet-500Bio, Microjet, Nagano, Japan), and 1 nl printing solution droplets were spotted and arrayed on it (Figure 1B, S1A). The coverslip was transferred to a humid chamber immediately after spotting and incubated for 2 h at 30°C, then washed with buffer C (0.1% SDS, 300 mM NaCl, 30 mM sodium citrate, pH 7.0) for 10 min to remove C-probes bound non-specifically to the glass surface and then rinsed with Milli-Q water three times. The spotted coverslips were dried in clean air and stored until use. A custom DNA microarray was used for quantification of miRNAs in total RNA prepared from human blood (Agilent Technologies, Tokyo, Japan). Designs of C-probes were 5′-(GTGCTTCATCTC-(ACA)_15_-AC)-3′ for miR-143, 5′-(AGTCTGATAAGCTA-(ACA)_15_-A)-3′ for miR-21, 5′-(TTTACGTGCTGCTA-(ACA)_15_-A)-3′ for miR-16, 5′-(GGACAAGTGCAATA-(ACA)_15_-A)-3′ for miR-92a and 5′-(TTTACACCCGGTGA-(ACA)_15_-A)-3′ for cel-miR-39. The 5′ ends of C-probes need to be phosphorylated for the LASH assay; therefore, C-probes, which were synthesized and immobilized on a DNA microarray via their 3′ ends, were incubated at room temperature for 90 min in buffer D [1 mM ATP, 0.25 units/µl T4 polynucleotide kinase, Takara 1x buffer (Takara, Tokyo, Japan)]. The phosphorylated custom DNA microarray was rinsed with Milli-Q water three times, then dried and stored until use.

### Fabrication of Hybridization Chamber Device

A hybridization chamber device was made of polydimethylsiloxane (PDMS; SILPOT, Dow Corning Toray, Tokyo, Japan). Sixteen button cell batteries (LR44, cylindrical, 1 mm in diameter, 5 mm in height) were arrayed 4×4 and immobilized on the bottom of a plastic box. Degassed SILPOT solution mix (184W: 184C = 10∶1) was then introduced into the box. After setting the PDMS at 80°C for 90 min, the hybridization chamber device was removed from the box. This chamber was used for the LASH assay (Figure S1).

### LASH Assay Protocol

First, 200 µl of the 1.5× hybridization buffer (100 mM Tris-HCl, pH 7.5, 15 mM MgCl_2_, 225 mM NaCl, 150 µM ATP, 15 mM DTT, 0.75 mg/ml BSA, 15% PEG5000) containing each 150 nM D-probe was mixed with 100 µl of various concentrations of the target miRNAs in RNase-free water. Three hundred microliters of this solution was incubated for 5 min at 95°C, and then cooled slowly to 30°C. After the addition of 4.3 µl 350 units/ml T4 DNA ligase, the solution was introduced into a chamber made of polydimethylsiloxane (PDMS) (Figure S1B) and capped by the coverslip, on which C-probes were immobilized. Next, the chamber was inverted and incubated for 2 h at 30°C on a shaker at 1,000 rpm. After incubation, the coverslip was peeled off from the chamber and washed twice with buffer E (30 mM NaCl, 3 mM sodium citrate, pH 7.0) for 10 min. It was dried in clean air and stored until fluorescence measurements were made.

### Measurement of Microarray Fluorescence Intensities

The coverslip, which was bound with target miRNAs and fluorescent probes, was placed on a fluorescence microscope (IX70, Olympus, Tokyo, Japan). 6-FAM-labeled C-probe, Alexa532-labeled D-probe and Alexa647-labeled D-probe were observed in the epi-illumination mode with a 50-W metal halide light source (U-LH50MH, Olympus, Tokyo, Japan) using the appropriate excitation band pass filter (BP460-490, Olympus, Tokyo, Japan for 6-FAM; D520/40M, Chroma Technology Corp., Bellows Falls, VT, USA for Alexa532; Semrock – FF01-628/40 BrightLine, IDEX Corp., Lake Forest, IL, USA for Alexa647). The emission light from the probes was collected by an objective lens (UPLANAPO 20×, N.A.0.70, Olympus, Tokyo, Japan) and captured by an electron-multiplying charge-coupled device (EM-CCD) camera (iXon3, Andor Technology Japan, Tokyo, Japan) using the appropriate emission band pass filter (HQ535/50 M, Chroma Technology Corp. for 6-FAM; Semrock - FF01-593/40 for Alexa532; Semrock – FF01-692/40 BrightLine, IDEX Corp. for Alexa647). Circular fluorescent probe spots (Figure 1C) in recorded images were enclosed by ellipsoidal regions of interest (ROIs) and the fluorescence intensities in ROIs were analyzed using NIH ImageJ software. Background intensities without C-probe were used for subtraction from the raw data.

### Cell Culture and Total miRNA Purification from Conditioned Medium

HEK293 cells, a human embryonic kidney cell line (CRL-1573) and HEK293 cells over-expressing miR-143 [28] were a kind gift from Dr. T. Ochiya (National Center Research Institute, Japan). Medium containing miRNAs released from HEK293 cells was collected as previously reported [28]. Briefly, these cell lines were cultured in Dulbecco’s modified Eagle’s medium (DMEM) (Life Technologies) with 10% heat-inactivated fetal bovine serum (FBS) at 37°C. Cells were then washed three times with Advanced DMEM (Life Technologies) containing penicillin, streptomycin and 2 mM L-glutamine in the absence of FBS (medium A), and then cultured in fresh medium A. After incubation for 72 h, medium A was collected and centrifuged at 2,000×*g* for 15 min and at 12,000 ×*g* for 35 min at room temperature. miRNAs were then extracted from the conditioned medium using the mirVana miRNA isolation kit (Life Technologies) according to the manufacturer’s protocol. In brief, 100 µl of conditioned medium or PBS buffer (137 mM NaCl, 2.7 mM KCl, 10 mM Na_2_HPO_4_, 1.76 mM KH_2_PO_4_, pH 7.4), was spiked with 1 µl of 1 nM synthesized miR-141. Two hundred microliters of the lysis/binding buffer provided in the kit was then added. After organic extraction and washing using the silica-based column provided in the kit, total miRNAs were eluted from the column by the addition of 100 µl 10 mM Tris-HCl buffer (pH 8.5). Synthesized miR-141 was also used as an external control for the conditioned medium (final concentration 10 pM) because the concentration of miR-141 in the medium was less than 15 fM.

### Quantification of miRNAs in Total RNAs Prepared from Human Blood

Two kinds of total RNAs (Human Adult Normal Tissue, Blood Vessel: [A] Artery, R1234013-10, Lot#A903111; [B] Vein, R1234020-10, Lot#A608254) were obtained from BioChain Institute Inc. (Newark, CA, USA). [A] and [B] samples are from an Asian male, aged 44 and a Caucasian female, aged 69, respectively. Total RNA and synthesized miRNA samples were spiked with cel-miR-39 from *C. elegans* as an external control and they were then analyzed by LASH assay or qRT-PCR. No cel-miR-39 signal could be detected in total RNA samples that had not been spiked. To correct for differences of hybridization yields in the LASH assay or reverse transcription yields in qRT-PCR between experiments, the fluorescence intensity and threshold cycle (Ct) value of each miRNA were normalized against those of cel-miR-39 for each experiment. On that basis, the fluorescence intensity and Ct value of each miRNA in total RNA samples were compared with those of synthesized miRNAs to estimate the amounts of miRNAs in total RNAs from human blood.

### Quantitative Reverse Transcription-Polymerase Chain Reaction (qRT-PCR)

cDNAs of purified miRNAs were generated using the TaqMan MicroRNA Reverse Transcription Kit (Life Technologies). Briefly, 5 µl purified total miRNA from HEK293 cells or total RNA from human blood was mixed with 7 µl master mix and 3 µl 5× RT primers, and then reverse-transcription reactions were performed on a thermal cycler according to the manufacturer’s protocol. To obtain standard curves, solutions containing synthesized miRNAs at various concentrations were measured in parallel. Using 3 µl cDNA solution, qRT-PCR was carried out in 96-well plates using the Applied Biosystems 7500 Fast Real-Time PCR System (Life Technologies). All reactions were performed in triplicate. The Ct value for each amount of input miRNA was determined automatically from amplification plots by the manufacturer’s software. The standard curves were fitted by a logarithmic equation, b-log(x)/log(1+a), where “a” indicates the amplification efficiency per cycle and “b” corresponds to the Ct value, for which input miRNA is 1 amol (Figure S2A, S3). The concentrations of miRNAs from biological samples were calculated based on their Ct values, and then they were normalized against those of the control miRNAs spiked in the samples.

## Results and Discussion

### Assay Design

In this study, miRNA quantification is based on the ligase-assisted sandwich hybridization (LASH) assay as depicted in Figure 1A. C-probe, which is complementary to part of the target miRNA, is immobilized on a glass surface via an ACA-repeat sequence and PEG linker. The flexible linkers are required for efficient hybridization of target nucleotides to a DNA probe immobilized on a solid substrate [29]. The target miRNA, D-probe, and C-probe hybridize to each other in the configuration shown in Figure 1A, and the three molecules are then combined covalently by T4 DNA ligase to prevent dissociation of the tertiary complex. D-probe has a stem-loop structure labeled with a fluorescent dye and a sequence complementary to the 3′ half of the miRNA. The stem-loop structure of D-probe is used to enable preferential binding to mature miRNAs and not to long pre-miRNAs [30]. More importantly, miRNA cannot be ligated with D-probe if D-probe does not have the stem-loop structure. In such a case, miRNA might release after the ligation of D-probe to C-probe and it could then hybridize to another C-probe and D-probe pair, resulting in multiple counting of the same miRNA. Thus, the stem-loop structure is needed for the LASH assay. In the LASH assay, miRNA does not release from C-probe and D-probe.

### Optimization of D-probe Complementary Sequence Length

Mature miR-143, 21 nucleotides in length, is down-regulated in prostate cancer and is, therefore, a potential biomarker of prostate cancer [31]. Thus, miR-143 was used as a model target miRNA to evaluate the LASH assay. First, we experimented with the length of both C-probe and D-probe to determine the optimum length. We prepared D-probe-143-(7) with seven nucleotides complementary to the 3′ end of the target miR-143 and C-probe-143-(14) with 14 nucleotides complementary to the 5′ end of the target. These were used as the D7 probe set. Also prepared were D-probe-143-(8), D-probe-143-(9) and D-probe-143-(10) with their appropriate C-probes and were defined as the D8 probe set, D9 probe set and D10 probe set, respectively (Figure 2A). Among the four probe sets, the D8 probe set showed the highest signal intensity of D-probe bound to miR-143 (Figure 2B).

### Sensitivity, Linearity and Specificity of the LASH Assay

Next, we investigated the dependence of signal intensity on input miRNA concentration using the D8 probe set. As shown in Figure 2C, the linearity of the plot is maintained at between 10 amol and 10 fmol (30 fM and 30 pM) of input miRNA, and the amount of bound D-probe-143-(8) reached saturation at 100 fmol. It is reasonable to assume that the upper limit of the linearity is dependent on the amount of C-probe immobilized on the substrate. In addition, a passenger strand of miR-143, which is produced with mature miR-143 from the miR-143 precursor, was used as a negative control. As expected, the signal of D-probe-143-(8) with the passenger strand was lower than the detection limit even in the presence of 10,000 fmol of the passenger strand, which indicated that the sensitivity for miR-143 is at least 10^6^ times greater than the sensitivity for the passenger strand. This result supports high sequence specificity in the LASH assay. On the other hand, the signal intensities without ligase were 50,000 times lower than those with ligase. These results indicate that T4 DNA ligase contributes to the high sensitivity of the LASH assay.

### Multi-color Detection of Homologous miRNAs

**Figure 3:**
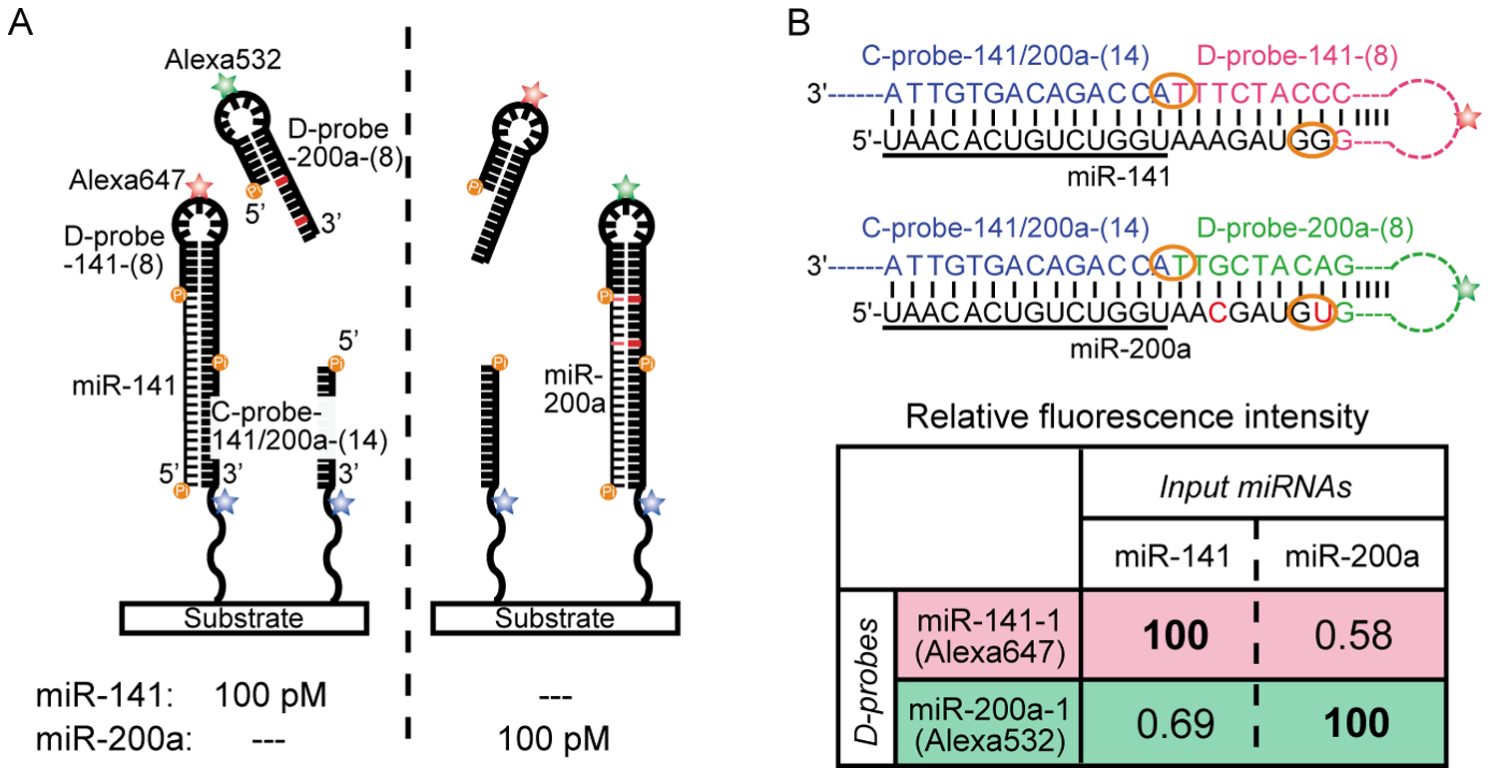
Distinction between homologous miRNAs in the LASH assay. (A) Schematic drawing of two-color detection experiments. Each D-probe can hybridize and be ligated to C-probe-141/200a-(14) in the presence of each target miRNA. (B) Complementary sequences of probe sets for miR-141 and miR-200a. Two nucleotides of the miR-200a sequence are different from those of miR-141 (red characters). These two nucleotides are located in the sequence that binds to the D-probe. On the other hand, the underlined sequences at the 5′ side of both miRNAs are identical and can bind to the common complimentary C-probe-141/200a-(14). The percentages of cross-hybridization of miR-141 and miR-200a to the opposite D-probes were evaluated.

Over 2,500 human miRNAs have been discovered, including many highly homologous miRNAs with almost the same sequence. The sandwich format assay has an inherent problem in distinguishing homologous sequences; namely if different nucleotide sequences exist in D-probes but not in C-probes for two homologous miRNAs, the two different D-probes will bind to the same immobilized C-probe. Thus, the sandwich format assay cannot distinguish such homologous miRNAs even though it can estimate the total amount of those homologous miRNAs. For example, miR-141 has been reported to be significantly up-regulated in blood vessels of prostate cancer patients [9]. However, miR-141 is highly homologous to miR-200a with only two 3′ nucleotide differences (Figure 3B). Therefore, both D-probe-141-(8) and D-probe-200a-(8) could bind to the common C-probe-141/200a-(14). There are two methods to avoid this problem. The first method is to swap the complementary sequence in D-probe for that in C-probe. If this method is applied to miR-141 and miR-200a, C-probe-141 becomes different from C-probe-200a because it is complementary to the 3′ side of miR-141, which has two nucleotides that are different from miR-200a. The second method involves multi-color detection using D-probes labeled with different fluorescent dyes. In this method, differently labeled D-probes bound to an identical C-probe can be separately analyzed according to each fluorescence wavelength. Here, we demonstrated two-color detection and investigated the cross-hybridization between homologous miRNAs. To distinguish miR-141 from miR-200a, Alexa532-labeled D-probe-200a-(8) and Alexa647-labeled D-probe-141-(8) were added together to the reaction. C-probe-141-(14) was used as the common C-probe-141/200a-(14) and immobilized on a substrate. In the presence of either 100 pM miR-141 or 100 pM miR-200a, signals of both Alexa532-labeled D-probe-200a-(8) and Alexa647-labeled D-probe-141-(8) were respectively measured using different emission filters (Figure 3A). The Alexa532-labeled D-probe-200a-(8) signal in the presence of miR-141 was 0.69% of the signal in the presence of miR-200a, while the signal of Alexa647-labeled D-probe-141-(8) in the presence of miR-200a was 0.58% of the signal with miR-141 (Figure 3B). These results indicate that each D-probe barely hybridizes to the non-homologous miRNA and multi-color detection enables the LASH assay to simultaneously quantify miR-141 and miR-200a without significant cross-hybridization.

### Quantification of Extracellular miR-143 Exported from HEK293 Cells

**Figure 4:**
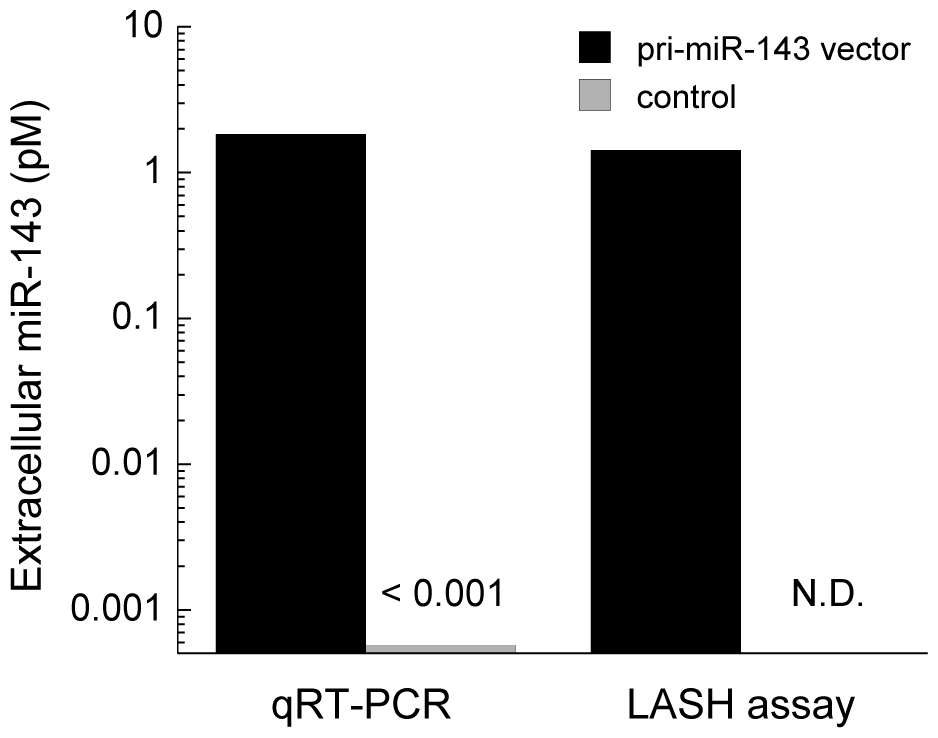
Quantification of extracellular miR-143 in total miRNA released from HEK293 cells using qRT-PCR or the LASH assay. The transfection of a primary miR-143 vector promoted the exocytosis of miR-143 from HEK293 cells compared with that from wild-type HEK293 cells, as described previously [28]. The LASH assay achieved the same quantification of miR-143 in total miRNA as qRT-PCR.

To examine if the LASH assay can quantify an endogenous miRNA in a total miRNA sample, we applied it to miRNAs exported from cultured cells. It has been reported that some miRNAs are released from HEK293 cells by exocytosis according to intracellular miRNA concentrations [28]. Because the normal HEK293 cell line expresses a small amount of miR-143 which is hardly detected, we prepared a HEK293 cell line engineered to over-express miR-143 in order to examine accurately whether qRT-PCR and LASH show the same result or not. We cultured normal HEK293 cells as well as HEK293 cells transfected with a vector containing pri-miR-143 (termed miR-143-HEK cells). Both cell lines were cultured for 3 days in the absence of FBS to prevent contamination of miRNAs derived from FBS. Total miRNAs were purified from the medium using a miRNA-purification kit and endogenous miR-143 was quantified by either qRT-PCR or LASH assay. Normal HEK293 cells released very low levels of miR-143 as described previously [28]. These levels were lower than the detection limit of the LASH assay (Figure 4). qRT-PCR was also unable to quantify miR-143 from normal HEK293 cells because the estimated miR-143 concentration was similar to background levels (0.0015 pM) (Figure S2B). On the other hand, miR-143-HEK cells produced much higher levels of extracellular miR-143; the concentration in the cell culture medium was estimated to be 1.84 pM by qRT-PCR and 1.42 pM by LASH assay. These results suggest that the LASH assay is able to quantify endogenous miRNA exported from living cells.

### LASH Assay of miRNAs in Total RNAs Derived from Blood

**Figure 5:**
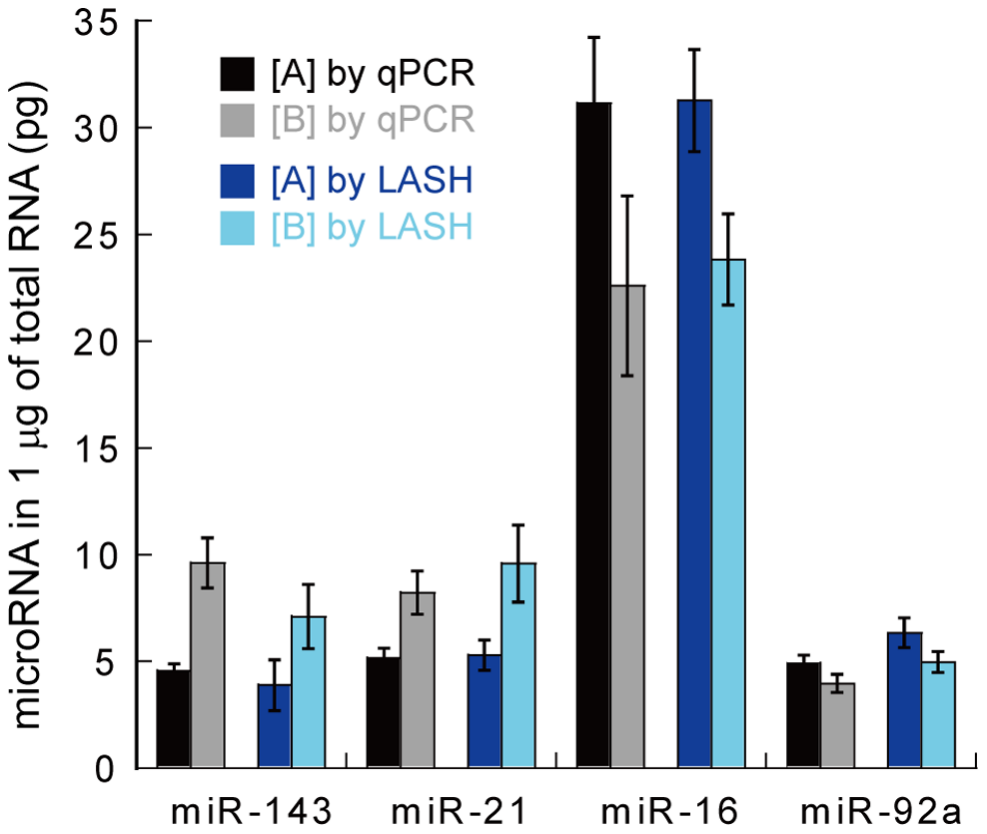
Quantification of miRNAs in total RNA samples derived from two types of human blood. The amounts of miRNAs in 1 µg of total RNA were estimated by qRT-PCR or LASH assay. The expression profiles of the two blood samples were reproducible between the two methods. Data represent the mean ± S.E. (n = 3).

To quantify miRNAs on a DNA microarray, we utilized a commercial custom DNA microarray from Agilent Corporation. miRNAs in two total RNA samples derived from different types of blood from healthy humans were quantified as described in Materials and Methods. The expression levels of miR-143, miR-21, miR-16 and miR-92a in each total RNA sample measured by LASH assay were similar to respective levels measured by qRT-PCR (Figure 5, Table S1). Correlation factors between qPCR and LASH assay were calculated to be 0.99 for [A] and 0.95 for [B] (Figure S4). This result indicates that the LASH assay is capable of quantifying miRNAs in total RNA using a commercial DNA microarray and of determining miRNA expression profiles.

This study demonstrated label-free quantification of miRNAs based on a DNA microarray approach, which has the potential for global profiling of miRNAs in biological samples, including blood. While conventional microarrays require additional pre-labeling and/or amplification steps, our LASH assay is free from such troublesome operations. Yan et al. have attempted to quantify miRNAs using a sandwich platform by enhancing sensitivity with gold nano particles and silver enhancing, but silver enhancing solution tends to be unstable and it is difficult to use as a commercial microarray platform [17]. Because the LASH assay does not require a signal amplification process, this simple method can be easily performed and combined with other technologies.

## Conclusions

We have developed a label-free and amplification-free quantitative assay of miRNAs on DNA microarrays. A ligase-assisted reaction coupled with a stem-loop D-probe enhances the sensitivity of miRNA to the probe 50,000-fold, enabling a sandwich format assay to quantify 30 fM (10 amol) of a short, 21 nucleotide RNA. This assay could be performed within 3 hours whereas conventional DNA microarrays for miRNA take whole a day. This label-free quantification method will be useful for other applications, including all-in-one microfluidic chips to diagnose diseases such as cancer.

## Supporting Information

Figure S1 Experimental procedures for the LASH assay using a DNA microarray. (A) Schematic configuration of probe-spots on a microarray. Printing buffer was spotted by an ink-jet machine on a circular area, 4.6 mm in diameter in a close packed configuration. One hundred twenty one spots were arrayed on the microarray. (B) The hybridization solution (300 µl) was introduced into the hybridization chamber and capped by a glass coverslip, on which capture probes were immobilized. The chamber was then inverted to bring the solution into contact with the probes and incubated for 2 h at 30°C on a shaker at 1,000 rpm.
(TIF)

Figure S2 qRT-PCR of miRNAs in cell culture medium. (A) Standard curves of synthetic miR-143 and miR-141 generated by qRT-PCR. The solid lines were calculated using the formula, b-log(x)/log(1+a), and are fitted to the data by least-squares fitting as described in Materials and Methods. (B) Raw concentration data for miR-143 and miR-141. Total miRNAs were purified from culture media collected from HEK293 cells, HEK293 cells transfected with primary miR-143 vector (HEK293*) or from PBS buffer only. The concentration of synthetic miR-141, which was spiked in all media before purification, was used to correct for any variability of miR-143 detection. Surprisingly, PBS buffer with miR-141 showed a very small miR-143 signal of ∼0.0015 pM. This is thought to be due to miR-143 contamination or/and cross-hybridization between spiked-in miR-141 and the TaqMan probes for miR-143. Therefore, we subtracted its value from raw miR-143 concentration data and we show the corrected concentrations in Figure 4.
(TIF)

Figure S3 Standard curves of synthetic cel-miR-39, miR-143, miR-21, miR-16 and miR-92a generated by qRT-PCR. The solid lines were calculated using the formula, b-log(x)/log(1+a), and are fitted to the data by least-squares fitting as described in Materials and Methods.
(TIF)

Figure S4 Correlation curves between qPCR and LASH for the blood assays. Black circles and gray circles indicate the amount of four kinds of miRNAs in sample [A] and [B], respectively. Correlation factors were calculated to be 0.99 for [A] and 0.95 for [B].
(TIF)

Table S1 Quantities of miRNAs in total RNAs prepared from human blood which were determined by qPCR and LASH.
(DOCX)
