# Supplementary figures and images for: Label-Free Quantification of MicroRNAs Using Ligase-Assisted Sandwich Hybridization on a DNA Microarray
Source: PLoS One. 2014 Mar 10;9(3):e90920. doi: 10.1371/journal.pone.0090920 (PMC3948704; doi:10.1371/journal.pone.0090920)

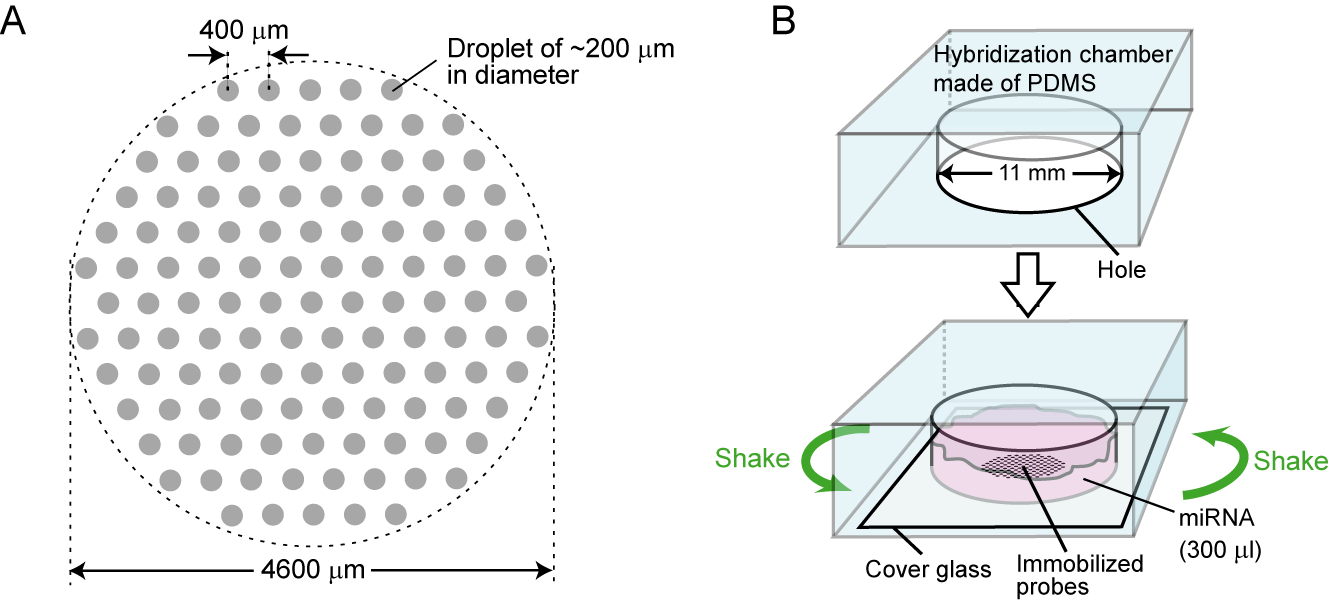

Supplement: Figure S1 — Experimental procedures for the LASH assay using a DNA microarray. (A) Schematic configuration of probe-spots on a microarray. Printing buffer was spotted by an ink-jet machine on a circular area, 4.6 mm in diameter in a close packed configuration. One hundred twenty one spots were arrayed on the microarray. (B) The hybridization solution (300 µl) was introduced into the hybridization chamber and capped by a glass coverslip, on which capture probes were immobilized. The chamber was then inverted to bring the solution into contact with the probes and incubated for 2 h at 30°C on a shaker at 1,000 rpm. (TIF) [file pone.0090920.s001.tif]

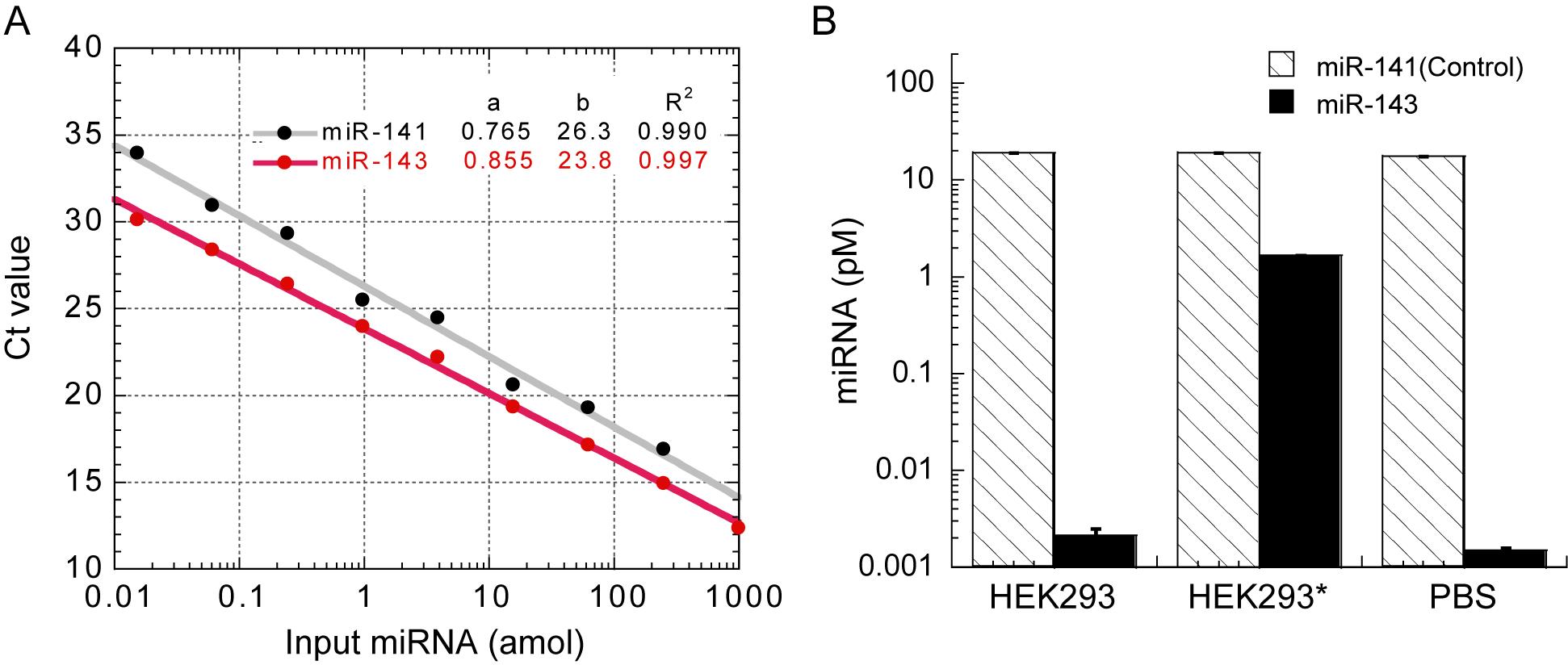

Supplement: Figure S2 — qRT-PCR of miRNAs in cell culture medium. (A) Standard curves of synthetic miR-143 and miR-141 generated by qRT-PCR. The solid lines were calculated using the formula, b-log(x)/log(1+a), and are fitted to the data by least-squares fitting as described in Materials and Methods. (B) Raw concentration data for miR-143 and miR-141. Total miRNAs were purified from culture media collected from HEK293 cells, HEK293 cells transfected with primary miR-143 vector (HEK293*) or from PBS buffer only. The concentration of synthetic miR-141, which was spiked in all media before purification, was used to correct for any variability of miR-143 detection. Surprisingly, PBS buffer with miR-141 showed a very small miR-143 signal of ∼0.0015 pM. This is thought to be due to miR-143 contamination or/and cross-hybridization between spiked-in miR-141 and the TaqMan probes for miR-143. Therefore, we subtracted its value from raw miR-143 concentration data and we show the corrected concentrations in Figure 4. (TIF) [file pone.0090920.s002.tif]

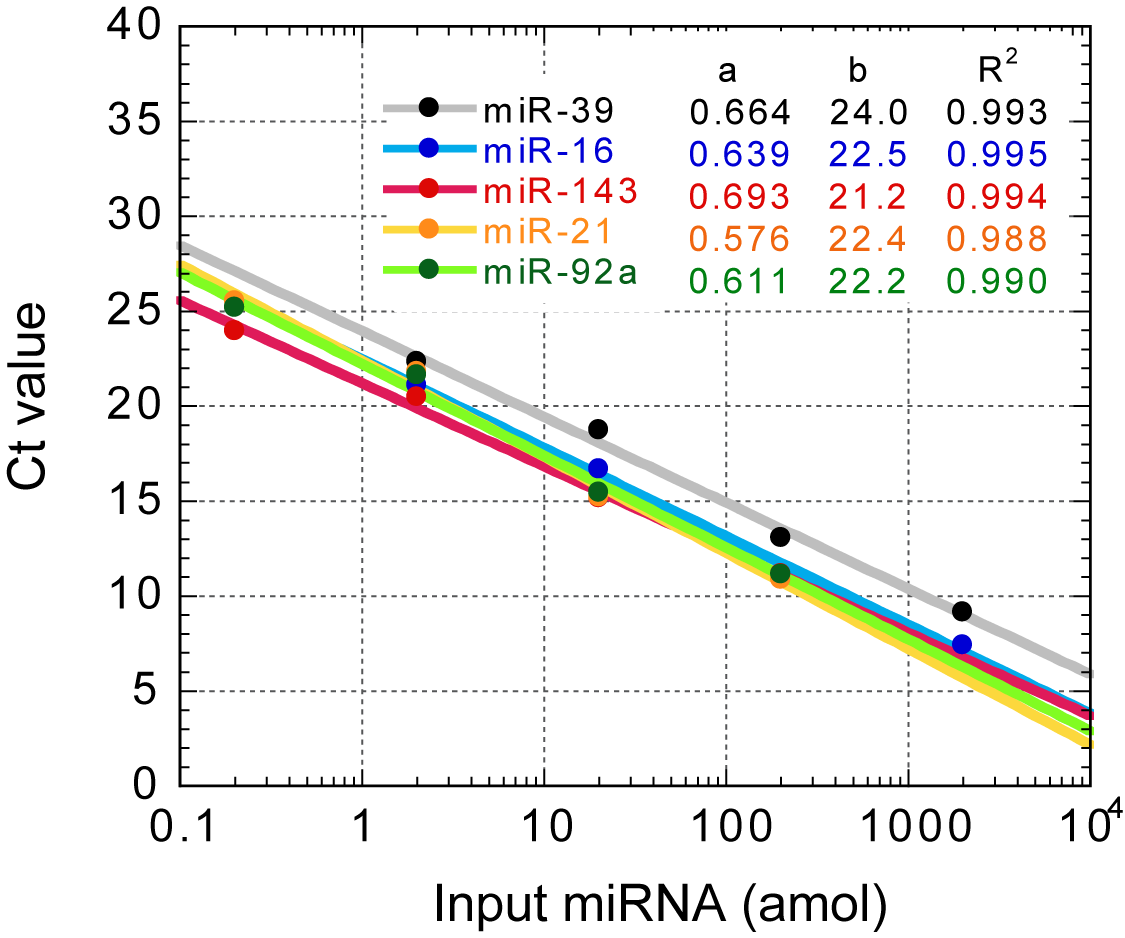

Supplement: Figure S3 — Standard curves of synthetic cel-miR-39, miR-143, miR-21, miR-16 and miR-92a generated by qRT-PCR. The solid lines were calculated using the formula, b-log(x)/log(1+a), and are fitted to the data by least-squares fitting as described in Materials and Methods. (TIF) [file pone.0090920.s003.tif]

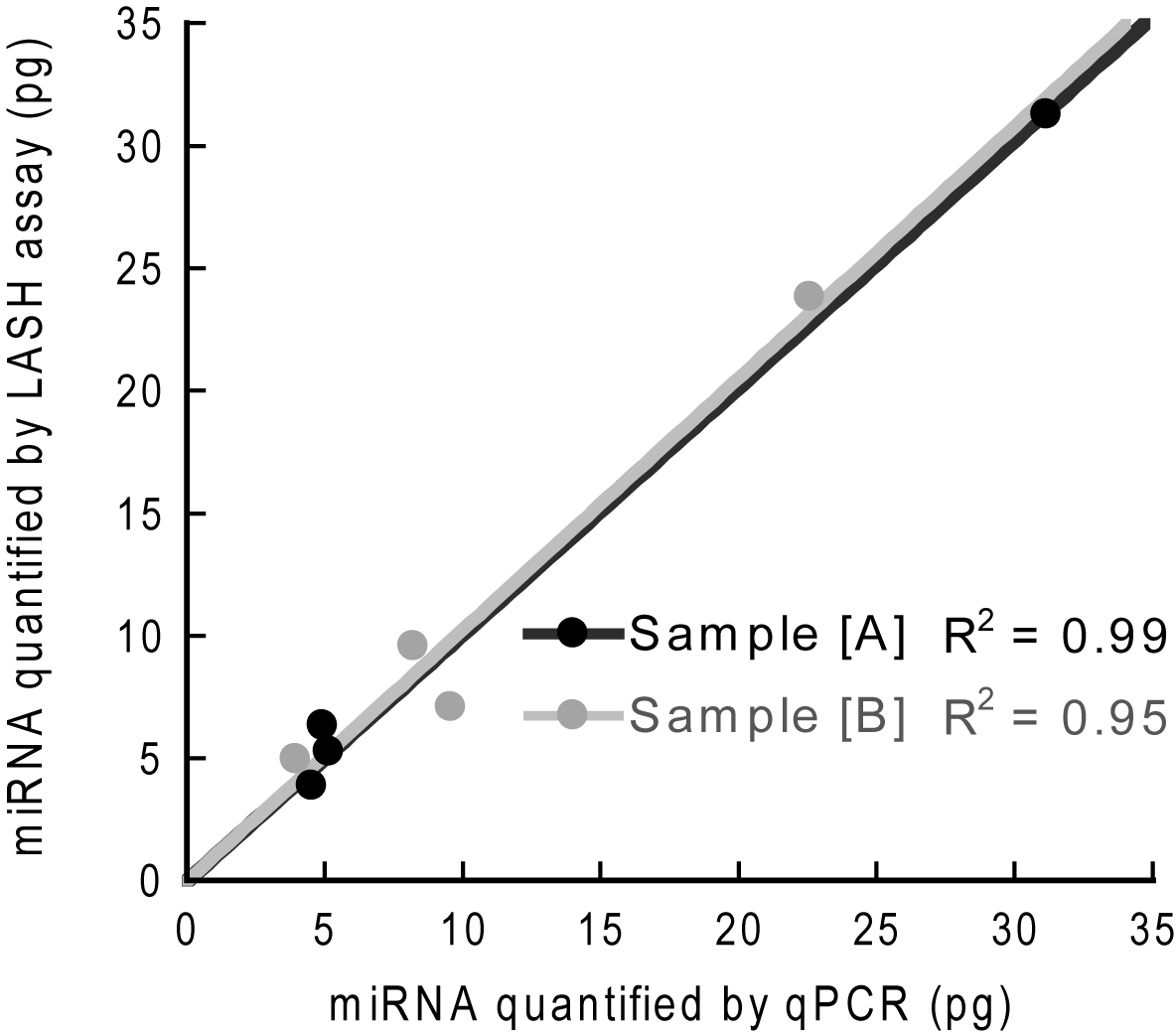

Supplement: Figure S4 — Correlation curves between qPCR and LASH for the blood assays. Black circles and gray circles indicate the amount of four kinds of miRNAs in sample [A] and [B], respectively. Correlation factors were calculated to be 0.99 for [A] and 0.95 for [B]. (TIF) [file pone.0090920.s004.tif]
