# Supplementary material for: Label-Free Quantification of MicroRNAs Using Ligase-Assisted Sandwich Hybridization on a DNA Microarray
Source: PLoS One. 2014 Mar 10;9(3):e90920. doi: 10.1371/journal.pone.0090920 (PMC3948704; doi:10.1371/journal.pone.0090920)
Supplement: Table S1 — Quantities of miRNAs in total RNAs prepared from human blood which were determined by qPCR and LASH. (DOCX) [file pone.0090920.s005.docx]

**Table S1.** Quantities of miRNAs in total RNAs prepared from human blood which were determined by qPCR and LASH. SEM means a standard error (n=3).

|  | qPCR | | | | LASH | | | |
| --- | --- | --- | --- | --- | --- | --- | --- | --- |
|  | [A] | | [B] | | [A] | | [B] | |
| miRNA | Average (pg) | SEM  (pg) | Average  (pg) | SEM  (pg) | Average  (pg) | SEM  (pg) | Average  (pg) | SEM  (pg) |
| miR-143 | 4.6 | 0.32 | 9.6 | 1.2 | 3.9 | 1.2 | 7.1 | 1.5 |
| miR-21 | 5.1 | 0.49 | 8.2 | 1.0 | 5.3 | 0.72 | 9.6 | 1.8 |
| miR-16 | 31 | 3.1 | 23 | 4.2 | 31 | 2.4 | 24 | 2.1 |
| miR-92a | 4.9 | 0.35 | 4.0 | 0.42 | 6.3 | 0.70 | 5.0 | 0.50 |
